# Machine learning for prediction of acute kidney injury in patients diagnosed with sepsis in critical care

**DOI:** 10.1371/journal.pone.0301014

**Published:** 2024-04-11

**Authors:** Jianshan Shi, Huirui Han, Song Chen, Wei Liu, Yanfen Li

**Affiliations:** 1 College of Biomedical Information and Engineering, Hainan Medical University, Haikou, R.P. China; 2 Interventional Vascular Surgery, The First Affiliated Hospital of Hainan Medical University, Haikou, P. R. China; 3 Department of Critical Medicine, Wanning People’s Hospital, Wanning, P. R. China; 4 Department of Infection, The First Affiliated Hospital of Hainan Medical University, Haikou, P. R. China; 5 Hainan Engineering Research Center for Health Big Data, Hainan Medical University, Haikou, P. R. China; Azienda Ospedaliero Universitaria Careggi, ITALY

## Abstract

**Background and objective:**

Acute Kidney Injury (AKI) is a common and severe complication in patients diagnosed with sepsis. It is associated with higher mortality rates, prolonged hospital stays, increased utilization of medical resources, and financial burden on patients’ families. This study aimed to establish and validate predictive models using machine learning algorithms to accurately predict the occurrence of AKI in patients diagnosed with sepsis.

**Methods:**

This retrospective study utilized real observational data from the Medical Information Mart for Intensive Care IV (MIMIC-IV) database. It included patients aged 18 to 90 years diagnosed with sepsis who were admitted to the ICU for the first time and had hospital stays exceeding 48 hours. Predictive models, employing various machine learning algorithms including Light Gradient Boosting Machine (LightGBM), EXtreme Gradient Boosting (XGBoost), Random Forest (RF), Decision Tree (DT), Artificial Neural Network (ANN), Support Vector Machine (SVM), and Logistic Regression (LR), were developed. The dataset was randomly divided into training and test sets at a ratio of 4:1.

**Results:**

A total of 10,575 sepsis patients were included in the analysis, of whom 8,575 (81.1%) developed AKI during hospitalization. A selection of 47 variables was utilized for model construction. The models derived from LightGBM, XGBoost, RF, DT, ANN, SVM, and LR achieved AUCs of 0.801, 0.773, 0.772, 0.737, 0.720, 0.765, and 0.776, respectively. Among these models, LightGBM demonstrated the most superior predictive performance.

**Conclusions:**

These machine learning models offer valuable predictive capabilities for identifying AKI in patients diagnosed with sepsis. The LightGBM model, with its superior predictive capability, could aid clinicians in early identification of high-risk patients.

## 1 Introduction

Sepsis, a prevalent critical condition in clinical practice [1–3], poses a significant risk for the development of Acute Kidney Injury (AKI) [4]. The kidneys are particularly vulnerable to reduced blood perfusion and certain treatment interventions, such as aggressive fluid resuscitation and mechanical ventilation, commonly employed in patients diagnosed with sepsis.

Currently, treatment for sepsis combined with AKI is mainly non-specific and lacks preventive measures. Studies have shown that the mortality rate of sepsis patients with AKI can increase from 38.2% to 70% [5, 6]. [7] reported that the incidence of AKI in sepsis patients is 40% to 50%, and the mortality rate after AKI increases 6-8 times, which is 7.79 times higher than non-sepsis patients. AKI is a common complication in ICU patients, with sepsis accounting for about 60% of cases, and 25% of patients requiring Continuous Renal Replacement Therapy (CRRT) treatment, resulting in longer hospital stays, higher mortality rates, and increased financial burden on families [8–10]. However, early identification and treatment of sepsis patients with AKI can promote early renal recovery, shorten hospital stays, and improve survival rates [11, 12]. Unfortunately, identifying high-risk AKI patients in the ICU is challenging for clinicians. Therefore, there is an urgent need to develop and promote reliable predictive models to identify these patients early and provide them with timely and effective interventions.

Despite advancements in medical treatments for AKI, the mortality rate associated with this condition remains unchanged [13]. Sepsis patients with AKI often experience multiple organ failure, microvascular dysfunction, and systemic inflammatory response syndrome, which further complicates clinical management [14–16]. However, early and effective interventions can potentially reverse AKI and reduce associated mortality rates [17]. Therefore, the identification of high-risk AKI patients among ICU patients diagnosed with sepsis is crucial. Improving early identification and preventive measures for AKI holds significant importance in enhancing the clinical outcomes of patients. Predicting AKI in sepsis patients has been a widely discussed topic in the field of critical care medicine [18].

Currently, numerous researchers are seeking widely applicable early AKI prediction methods. Several biomarkers have been reported to be associated with AKI in sepsis, including procalcitonin [19], microRNA-22-3p [20], neutrophil gelatinase-related lipoproteins [21], urine miR-26b [22], soluble thrombomodulin [23], Tissue Inhibitor of Metalloproteinase-2(TIMP-2), and Insulin-like Growth factor-Binding Protein 7 (IGBP-7) [24]. However, the high requirements and costs associated with these biomarker detection technologies hinder their clinical applicability. Several scoring systems, such as the Simplified Acute Physiology Score (SAPS-II), the Acute Physiology and Chronic Health Score II (APACHE-II), and the Sequential Organ Failure Assessment (SOFA), are also utilized for AKI prediction. However, these scoring systems have shown poor specificity and sensitivity in predicting AKI in patients diagnosed with sepsis, leading to unsatisfactory results [25, 26].

To address the limitations mentioned above, researchers have proposed the use of multivariate predictive models based on traditional statistical methods to predict the progression of AKI in sepsis patients. For instance, [27] developed a predictive model for AKI in sepsis patients using Logistic Regression (LR) and COX proportional hazard model. While this model demonstrated good prediction accuracy, LR assumes linear correlations between independent and dependent variables, which may oversimplify the complex nonlinear relationships and lead to decreased model performance. Therefore, there is a need to explore more efficient and accurate predictive tools for managing sepsis patients. With advancements in computer technology and statistical theory, machine learning has gained attention from clinical practitioners as a potential solution for this purpose [28].

The LR model assumes a linear relationship between features and outputs, while machine learning algorithms like Light Gradient Boosting Machine (LightGBM), Extreme Gradient Boosting (XGBoost), Support Vector Machines (SVM), Artificial Neural Networks (ANN), Decision Trees (DT), and Random Forests (RF) offer the capacity for automatic learning from data [29]. These machine learning models can capture non-linear correlations and interactions, allowing for the exploration of more complex relationships between features through intricate model structures and algorithms, making them more flexible [30]. Therefore, machine learning algorithms present a promising opportunity to enhance the prediction of AKI in septic patients.

Machine learning technology has emerged as a promising approach for disease prediction models. Within the realm of AKI prediction, researchers have diligently explored the potential of machine learning algorithms. For instance, [31] developed an automated continuous random prediction algorithm for AKI, showcasing its efficacy in early identification of high-risk patients. Additionally, [32] conducted a comparative study among SVM, ANN, RF, and Simplified Acute Physiology Score II (SAPSII) in predicting AKI patient mortality, where RF exhibited superior potential. [33] extracted patient data from the Medical Information Mart for Intensive Care III (MIMIC-III) database, specifically focusing on patients diagnosed with Congestive Heart Failure (CHF). Several common machine learning classifiers were compared to select the optimal predictive model for identifying CHF patients at high risk of developing AKI. Among these classifiers, the LightGBM model demonstrated the best predictive performance, achieving an Area Under the Receiver Operating Characteristic Curve (AUC) of 0.80313. However, the optimal machine learning model for predicting AKI in septic patients remains unclear. This study aims to develop and validate an AKI predictive model using machine learning technologies and compare the performance of different models to determine the most effective one.

## 2 Materials and methods

### 2.1 Data source

The sepsis patient data utilized in this study originated from Medical Information Mart for Intensive Care IV (MIMIC-IV) version 2.0 [34], which were launched on June 12, 2022, as a publicly accessible database from a single center. MIMIC-IV is a publicly available relational dataset set up by the Laboratory for Computational Physiology that includes deidentified health data related to thousands of intensive care unit admissions, including real hospital stays for patients enrolled to a tertiary academic medical center in Boston, MA, USA. MIMIC-IV includes comprehensive data for every patient while they are in the hospital: laboratory measurements, medications managed, vital signs documented. Extra data available contains the out-of-hospital date of death, data from the online medical record system (which provides for height and weight), and more information for continuous infusions in the ICU. The dataset is extensively adopted by inquirers and engineers in the world, so as to promote studies in clinical informatics, epidemiology, and machine learning.

#### Ethical statement

This research utilized data from the MIMIC-IV database. Given that the MIMIC IV database consists of de-identified data and is publicly accessible, the study was not subject to specific ethical review. However, we conducted the research in accordance with fundamental ethical principles and ensured the validity and fairness of the study process.

### 2.2 Participants

**Fig 1:**
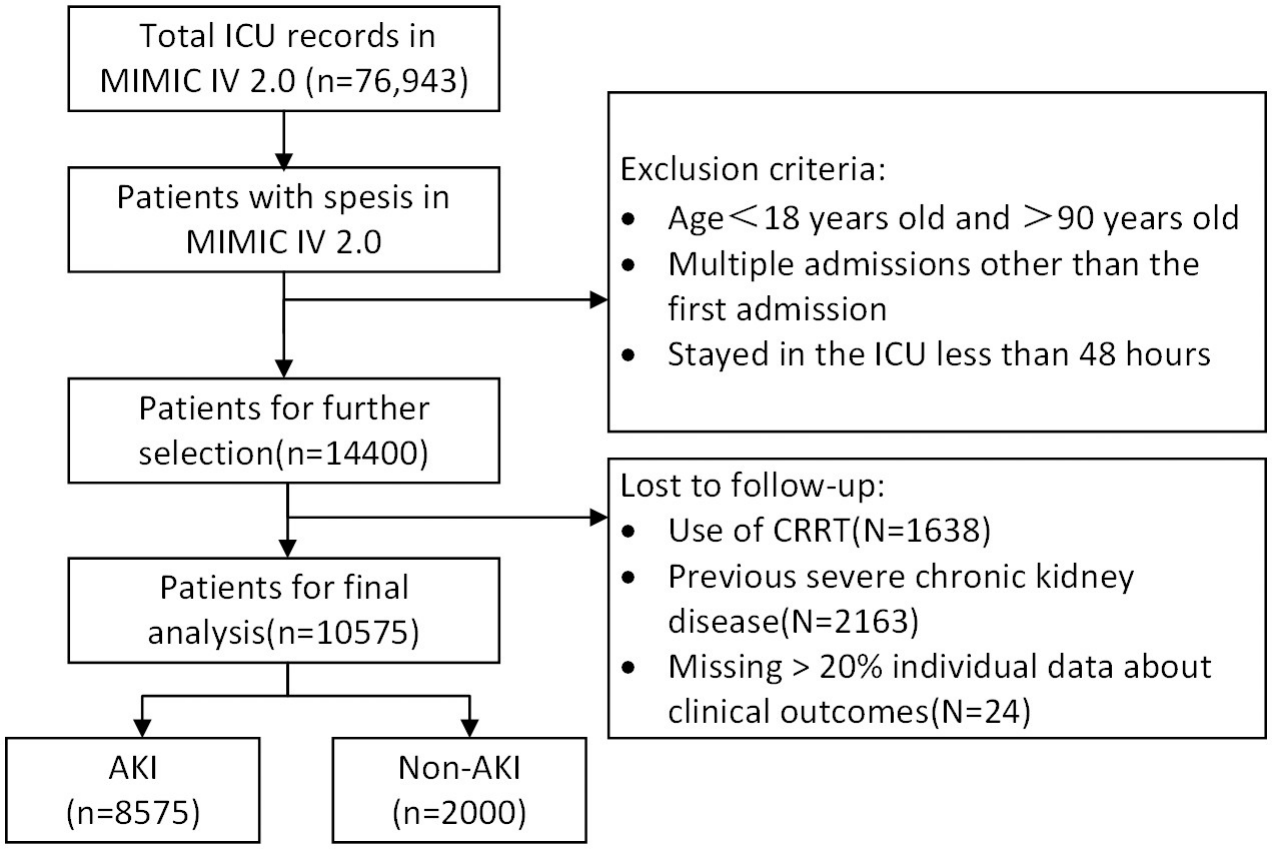
Flow chart of patients’ selection.

When an infection occurred and patients’ SOFA score ≥ 2, the patients qualification were taken into account. The occurrence of AKI among patients diagnosed with sepsis during admission were assessed using the Kidney Disease Improving Global Outcomes (KDIGO) standards [35]. Patients who stayed in the ICU less than 48 hours, aged < 18 years old and > 90 years old, or multiple admissions other than the first admission old were excluded. Additionally, patients who received CRRT and had a history of severe chronic kidney disease on individual clinical outcomes were also excluded. Furthermore, variables with more than 20% missing values were excluded to minimize bias resulting from missing data. Missing values were handled using median imputation, a method that involves replacing missing values with representative measures of central tendency to maintain the general distributional shape and characteristics of the original data. The final cohort consisted of 10,575 patients, of whom 8,575 developed AKI, while 2,000 did not. Within this cohort, 1,747 patients experienced septic shock, 13 individuals faced severe hypoxemia, and 9,960 required ventilation. On average, the time between the onset of sepsis and ICU admission was 1.04 days. Fig 1 shows the flow chart of patients’ selection.

### 2.3 Variable selection

With the help of PostgreSQL 13, we installed the database on the service, and then extracted the demographic, clinical characteristics, etc., of sepsis patients according to the corresponding SQL codes. The MIMIC-IV database provided patient information in the initial 48 hours after admission. The data below were adopted in this research:

1. Demographic features, including age, gender and race;
2. Characteristics of hospitalization, including admission type and first care unit;
3. Vital signs, including temperature, Heart Rate(HR), Respiratory Rate(RR), Systolic Blood Pressure (SBP), Diastolic Blood Pressure (DBP), Mean Arterial Pressure(MAP), oxygen saturation(SpO2);
4. Laboratory parameters, including PH value, pao2, paco2, hco3, base excess, potassium, sodium, chloride, magnesium, calcium, phosphate, White Blood Cell count(WBC), Red Blood Cell count(RBC), platelets, hemoglobin, hematocrit, glucose, Blood Urea Nitrogen (BUN), creatinine, Prothrombin Time(PT), International Normalized Ratio (INR), Partial Thromboplastin Time (PTT), and urine volume on the first day;
5. Comorbidities, including myocardial infarct, congestive heart failure, cerebrovascular disease, diabetes, peptic ulcer disease, severe liver disease, malignant cancer, Acquired Immune Deficiency Syndrome (AIDS), Coronary Artery Disease (CAD), Atrial Fibrillation(AFib), and acute respiratory distress syndrome (ARDS);
6. Rating scales, including Oxford Acute Severity of Illness Score (OASIS).

Table 1 lists the details of variables definitions.

**Table 1:** Variables definitions.

Characteristics	Name	Unit	Type	Description
Demographic features	Age	-	Continuous	Years
	Gender	-	Categorical	Female, Male
	Race	-	Categorical	BLACK, WHITE, ASIAN, etc.
Characteristics of hospitalization	Admission type	-	Categorical	ELECTIVE, URGENT, DIRECT EMER, etc.
	First care unit	-	Categorical	CCU, SICU, MICU, etc.
Vital signs	Temperature max	°C	Continuous	Maximum on day 1 after ICU
	Heart rate max	bpm	Continuous	Maximum on day 1 after ICU
	Resp rate max	insp/min	Continuous	Maximum on day 1 after ICU
	SBP min	mmHg	Continuous	Minimum on day 1 after ICU
	DBP min	mmHg	Continuous	Minimum on day 1 after ICU
	MAP min	mmHg	Continuous	Minimum on day 1 after ICU
	Spo2 min	%	Continuous	Minimum on day 1 after ICU
Laboratory parameters	PH	-	Continuous	First value on day 1 after ICU
	Pao2	mm Hg	Continuous	First value on day 1 after ICU
	Paco2	mm Hg	Continuous	First value on day 1 after ICU
	Hco3	mEq/L	Continuous	First value on day 1 after ICU
	Base excess	mEq/L	Continuous	First value on day 1 after ICU
	Potassium	mEq/L	Continuous	First value on day 1 after ICU
	Sodium	mEq/L	Continuous	First value on day 1 after ICU
	Chloride	mEq/L	Continuous	First value on day 1 after ICU
	Magnesium	mg/dL	Continuous	First value on day 1 after ICU
	Calcium	mg/dL	Continuous	First value on day 1 after ICU
	Phosphate	mg/dL	Continuous	First value on day 1 after ICU
	WBC	K/uL	Continuous	First value on day 1 after ICU
	RBC	m/uL	Continuous	First value on day 1 after ICU
	Platelets	K/uL	Continuous	First value on day 1 after ICU
	Hemoglobin	g/dL	Continuous	Mean value on day 1 after ICU
	Hematocrit	%	Continuous	First value on day 1 after ICU
	Glucose	mg/dL	Continuous	First value on day 1 after ICU
	BUN	mg/dL	Continuous	First value on day 1 after ICU
	Creatinine	mg/dL	Continuous	First value on day 1 after ICU
	PT	sec	Continuous	First value on day 1 after ICU
	INR	%	Continuous	First value on day 1 after ICU
	PTT	sec	Continuous	First value on day 1 after ICU
	First day urine output	ml	Continuous	First value on day 1 after ICU
Comorbidities	Myocardial infarct	-	Categorical	1/0
	Congestive heart failure	-	Categorical	1/0
	Cerebrovascular disease	-	Categorical	1/0
	Diabetes	-	Categorical	1/0
	Peptic ulcer disease	-	Categorical	1/0
	Severe liver disease	-	Categorical	1/0
	Malignant cancer	-	Categorical	1/0
	AIDS	-	Categorical	1/0
	CAD	-	Categorical	1/0
	AFIB	-	Categorical	1/0
	ARDS	-	Categorical	1/0
Rating scale	OASIS	-	Continuous	Maximum on day 1 after ICU

### 2.4 Prediction model

This research compared several machine learning models, namely LightGBM, XGBoost, RF, DT, ANN, SVM, and LR. Both LightGBM and XGBoost, as boosting algorithms, offer distinct advantages in scenarios with limited training samples, short training times, and limited reference knowledge. Among these models, LightGBM exhibited superior performance, a point we will delve into further. LightGBM, an open-source gradient boosting framework, extends upon the gradient boosting framework of DT. This framework enhances model efficiency and reduces memory usage. LightGBM finds common usage in ranking, classification, and various machine learning tasks. It integrates two key technologies: Gradient-based One-Side Sampling (GOSS) and Exclusive Feature Bundling (EFB), enabling faster algorithm execution while maintaining high precision.

**(1) GOSS Technique for LightGBM**. Different data instances have varying impacts on data gain calculation. Instances with larger gradients contribute more to data gain. GOSS eliminates instances with smaller gradients while retaining those with larger gradients to maintain data gain estimation accuracy, resulting in more precise information gain estimates with smaller datasets. In GOSS, prediction values (preds) and LOSS are computed. Training examples are sorted based on their absolute values in descending order. Assuming constant factors *a* and *b*, we retain the top *x* × 100% instances to form the subset ‘topSet’, and sample the smaller gradient instances at *y* × 100% to create the subset ‘randSet’. It’s crucial to amplify the gradient of the small gradient sample by (1 − *x*)/*y*. Algorithm 1 outlines this process.

**Algorithm 1** Gradient-based One-Side Sampling.

**Input:** td: training data, it: iterations

**Input:** x: sampling proportion of big gradient data

**Input:** y: sampling proportion of small gradient data

**Input:** loss: loss function, L:week learner

*models* ← {}, $fact\leftarrow \frac{1-x}{y}$

*topN* ← *x* × *len*(*td*), *randN* ← *y* × *len*(*td*)

 **for** *i* = 1 to *it* **do**

  *fores* ← *models*.*predictive*(*td*)

  *g* ← *loss*(*td*, *fores*), *w* ← 1, 1, …

  *sort* ← *GetSortedIndices*(*abs*(*g*))

  *topnSet* ← *sort*[1 : *topN*]

  *randomSet* ← *RandomPick*(*sort*[*topN* : *len*(*td*)], *randomN*)

  *useSet* ← *topnSet* + *randomSet*

  *w*[*randomSet*] × = *fact* ⊳ *Assignweightfacttothesmallgradientdata*.

  *newModel* ← *L*(*td*[*useSet*], −*g*[*useSet*], *w*[*useSet*])

  *models*.*append*(*newModel*)

 **end for**

**(2) EFB Technique for LightGBM**. The EFB algorithm employs a graph-based approach, treating each characteristic as a node within the structure. It connects unique features based on their relationships, forming bundled feature sets within the graph. The greedy strategy for the EFB characteristic bundling involves the following steps:

1. Using the features as vertices in the graph, connect unique features (samples existing non-zero simultaneously), with the number of coexisting non-zero samples as the edge weight.
2. Sort the features based on vertex degree, indicating higher degrees represent greater conflicts among characteristics (less likely to bind with other features).
3. Establish a maximum conflict threshold, *K*. Begin an outer loop to iterate through the sorted features. For each feature, traverse existing feature bundles. If the feature fits within a cluster, add it. If not, create a new cluster and add the feature.

Algorithm 2 describes the algorithm process.

**Algorithm 2** Greedy Bundling.

**Input:** C: characteristic, M: max conflict count

*BuildgraphG*

*sortOrder* ← *G*.*sortByDegree*()

*bundles* ← {}, *bunConflict* ← {}

 **for** *i* in *sortOrder* **do**

  *needN* ← *True*

  **for** *j* = 1 to *len*(*bundles*) **do**

   *conflict* ← *ConflictCnt*(*bundles*[*j*], *C*[*i*])

   **if** *conflict* + *bunConflict*[*i*] ≤ *M* **then**

    *Bundles*[*j*].*add*(*C*[*i*]), *needN* ← *False*

    Break

   **end if**

  **end for**

  **if** needN **then**

   Add *C*[*i*] to bundles as a new bundle

  **end if**

 **end for**

**Onput:** bundles

## 3 Results

### 3.1 Study population

In the final cohort, 10,575 patients were included, of which 8,575 cases (81.1%) developed AKI during hospitalization in the MIMIC-IV database. A total of 47 variables were selected for model construction. To determine the predictive power of different features, the collections and distributions of categorical variables were first analyzed by performing a chi-squared test (*p* < 0.05). Table 2 lists the statistical values of the features of the detailed patient demographic data with their corresponding definitions.

**Table 2:** Characteristics between AKI and non-AKI groups of the patients diagnosed with sepsis.

Characteristics	Total	Non-AKI	AKI	P-Value
	(n = 10575)	(n = 2000)	(n = 8575)	
Demographic variables				
Male, n(%)	6006(56.8%)	1112(56%)	4894(57%)	0.0000
Age (mean (SD))	66.1(16.5)	61.5(16.0)	67.0(18.5)	0.0000
Ethnicity, n (%)				
White	6930(65.5%)	1247(62.4%)	5683(66.3%)	0.0000
Black	725(6.9%)	154(7.7%)	571(6.7%)	0.0000
Other	2920(27.6%)	599(30.0%)	2321(27.1%)	0.0000
Vital signs variables, mean (SD) if not otherwise specified				
Temperature max(°C)	37.58(0.85)	37.66(0.82)	37.57(0.86)	0.0000
Heart rate max (bpm)	107.51(21.70)	107.64(21.41)	107.48(21.75)	0.7520
Resp rate max (insp/min)	28.69 (6.70)	28.62 (6.85)	28.71 (6.67)	0.5430
SBP min (mmHg)	88.11 (16.25)	91.58 (16.22)	87.46 (16.17)	0.0000
DBP min (mmHg)	44.74(10.72)	46.89 (10.78)	44.34 (10.66)	0.0000
MAP min (mmHg)	56.39(13.53)	59.01(13.01)	55.90(13.57)	0.0000
Spo2 min (%)	91.51(6.56)	92.22(5.39)	91.38(6.74)	0.0000
Laboratory variables, mean (SD) if not otherwise specified				
PH	7.35(0.11)	7.36(0.10)	7.35(0.11)	0.0000
Pao2 (mmhg)	168.90(136.77)	157.87(136.74)	170.58(136.69)	0.0004
Paco2 (mmhg)	43.58(13.93)	42.43(13.33)	43.75(14.01)	0.0003
Hco3 (meq/L)	84.08(19.53)	83.72(16.95)	84.12(19.84)	0.0061
Baseexcess (meq/L)	1.73(5.74)	1.30(5.27)	1.79(5.80)	0.0010
Potassium (meq/L)	4.32(0.88)	4.18(0.85)	4.34(0.89)	0.0000
Sodium (meq/L)	138.22(5.87)	138.26(6.00)	138.22(5.84)	0.7360
Chloride (meq/L)	103.39(7.32)	103.45(7.40)	103.38(7.30)	0.6611
Magnesium (mg/dL)	2.00(0.54)	1.90(0.41)	2.02(0.55)	0.0000
Calcium (mg/dL)	8.29(0.99)	8.29(1.09)	8.30(0.97)	0.7257
Phosphate (mg/dL)	3.86(1.57)	3.42(1.32)	3.94(1.60)	0.0000
WBC (k/uL)	13.59(11.20)	13.62(15.41)	13.59(10.22)	0.8914
RBC (m/uL)	3.67(0.85)	3.72(0.82)	3.66(0.85)	0.0011
Platelets (k/uL)	213.64(118.45)	216.05(116.60)	213.19(118.78)	0.2803
Hemoglobin (g/dL)	11.00(2.48)	11.18(2.42)	10.97(2.49)	0.0002
Hematocrit (%)	33.49(7.37)	33.73(7.06)	33.45(7.43)	0.0868
Glucose (Mg/Dl)	155.03(89.87)	148.06(91.28)	156.33(89.54)	0.0000
BUN (mg/dL)	29.16(23.76)	25.37(23.03)	29.86(23.82)	0.0000
Creatinine	1.56(1.60)	1.24(1.20)	1.62(1.66)	0.0000
PT (sec)	15.86(8.30)	15.33(8.10)	17.16(11.15)	0.0000
INR	16.89(10.76)	1.40(0.83)	1.58(1.09)	0.0000
PTT (sec)	1.55(1.06)	33.62(17.76)	38.11(23.34)	0.0000
First day urine output	13918.57(17021.43)	2540.85(1450.23)	1636.64(1208.74)	0.0000
Comorbidities, n (%)				
Myocardial infarct	1592(15.05)	197(9.85)	1395(16.27)	0.0000
Congestive heart failure	2482(23.47)	275(13.75)	2207(25.74)	0.0000
Cerebrovascular disease	1885(17.83)	345(17.25)	1540(17.96)	0.2285
Diabetes	2605(24.63)	389(19.45)	2216(25.84)	0.0000
Peptic ulcer disease	318(3.01)	63(3.15)	255(2.97)	0.3386
Severe liver disease	682(6.45)	86(4.30)	596(6.95)	0.0000
Malignant cancer	1439(13.61)	284(14.20)	1155(13.47)	0.1949
AIDS	77(0.73)	28(1.40)	49(0.57)	0.0000
CAD	3657(34.58)	451(22.55)	3206(37.39)	0.0000
AFIB	3657(34.58)	451(22.55)	3206(37.39)	0.0000
ARDS	4267(40.35)	549(27.45)	3718(43.36)	0.0000
Rating scale, mean (SD) if not otherwise specified				
OASIS	12.44(8.08)	31.56(7.60)	37.67(9.00)	0.0000

### 3.2 Model performance comparisons

Experimental comparisons were conducted on a 64-bit Windows 10 system equipped with Python 3.11 (www.python.org). The computer features an Intel(R) Core (TM) i5-6300U CPU running at 2.40GHz and 16.00 GB RAM. Regarding the predictive model construction, 80% of the cases were randomly allocated to the training set, while the remaining 20% of cases were reserved for the testing set.

First, the outcomes of these 7 machine learning approaches are shown in Table 3. The LightGBM model exhibited superior performance compared to other models, achieving the highest scores in accuracy (0.829), F1 score (0.902), and AUC (0.801). Accuracy measures the proportion of correctly predicted samples, the F1 score combines precision and recall, while AUC assesses the model’s ability to classify positive and negative samples. Across these metrics, LightGBM consistently outperformed other models, indicating its superior overall performance and stronger predictive capabilities in this classification task, ultimately leading to more accurate sample classification. On the other hand, SVM exhibits the highest specificity and precision, indicating its capability to accurately classify negative instances and correctly predict positive instances, respectively. Meanwhile, DT shows the highest sensitivity, excelling in the correct identification of true positive instances. For the prediction of AKI, it is premised on the need for accurate diagnosis of AKI in patients diagnosed with sepsis. Therefore, sensitivity ought to be taken into consideration. Although the DT model achieved highest sensitivity (0.987), it obtained least precision (0.816). To take into account both precision and recall, F1 score providing a balance between precision and recall is regarded as a metric. Overall, each model showcases distinct strengths, with LightGBM leading in overall performance, while SVM and DT excel in certain specific metrics. When moving towards real-world applications, it would be prudent to factor in specific requirements and focal points.

**Table 3:** Comparing the performance of the 7 models.

	LightGBM	XGBoost	RF	DT	ANN	SVM	LR
Accuracy	**0.829**	0.824	0.815	0.809	0.781	0.728	0.821
Specificity	0.219	0.246	0.102	0.050	0.328	**0.614**	0.152
Sensitivity	0.973	0.960	0.982	**0.987**	0.887	0.754	0.978
Precision	0.841	0.844	0.823	0.816	0.849	**0.893**	0.831
F1	**0.902**	0.898	0.896	0.893	0.868	0.818	0.898
AUC	**0.801**	0.773	0.772	0.737	0.720	0.765	0.776

**Fig 2:**
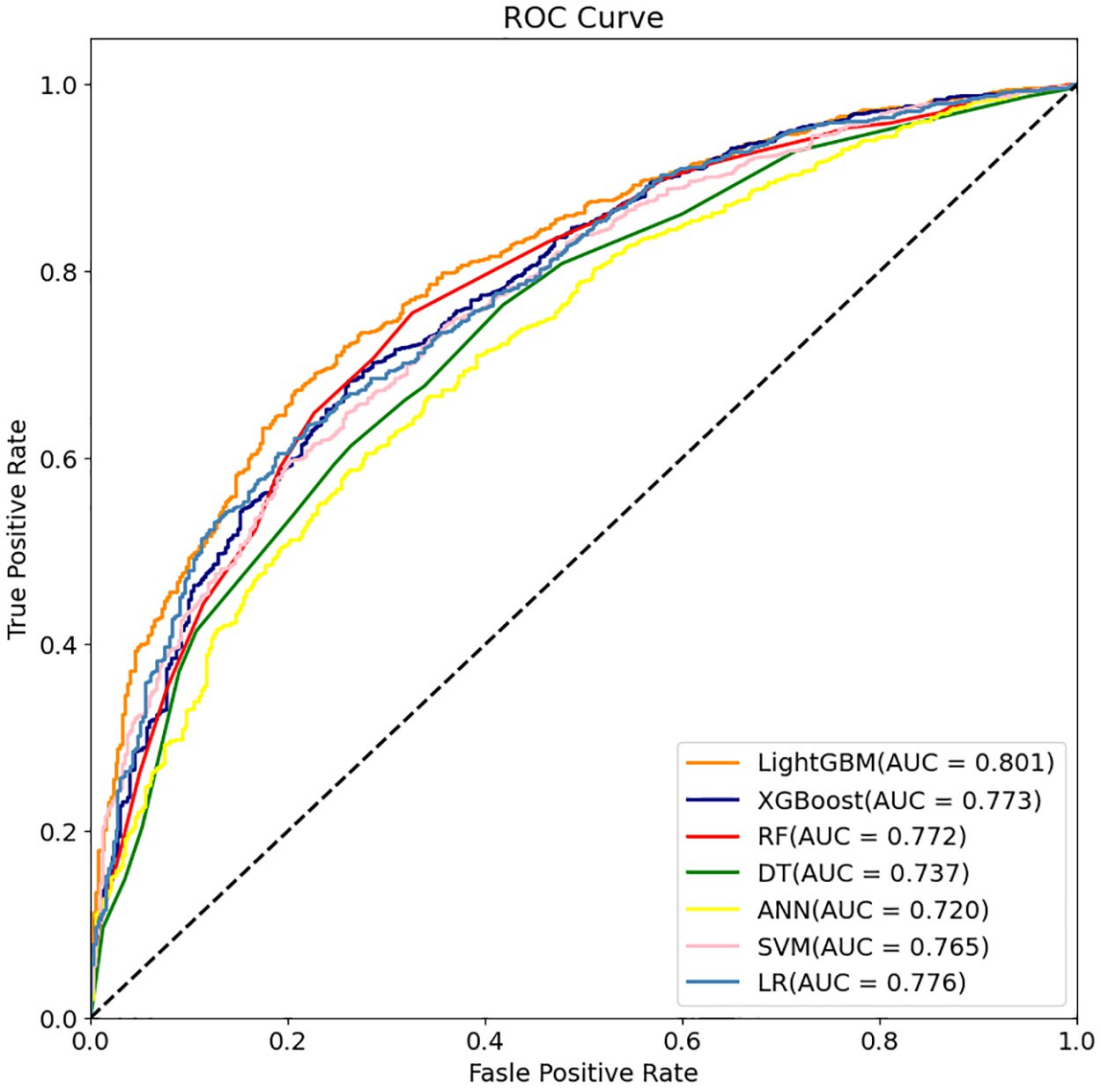
Comparison of the ROC curve of the 7 models. ROC = Receiver Operating Characteristic, TPR = True Positive Rate, TNR = True Negative Rate, AUC = Area Under the ROC Curve.

Then, the evaluation then proceeds to assess and explore the models’ performance concerning the AUC. As depicted in Fig 2, the Receiver Operating Characteristic (ROC) curve illustrates the predictive models’ performance. Fig 2 demonstrates that LightGBM exhibits a notably higher AUC compared to the other models. While LR, XGBoost, SVM, and RF’s ROC curves demonstrate better performance than DT and ANN, they still fall short of LightGBM’s performance. Among these seven models, LightGBM’s ROC curve attains the highest point, indicating superior classification performance. Specifically, this model achieves the highest AUC, signifying a favorable balance between sensitivity (True Positive Rate, TPR) and specificity (True Negative Rate, TNR), achieving a higher true positive rate while maintaining a lower false positive rate.

**Fig 3:**
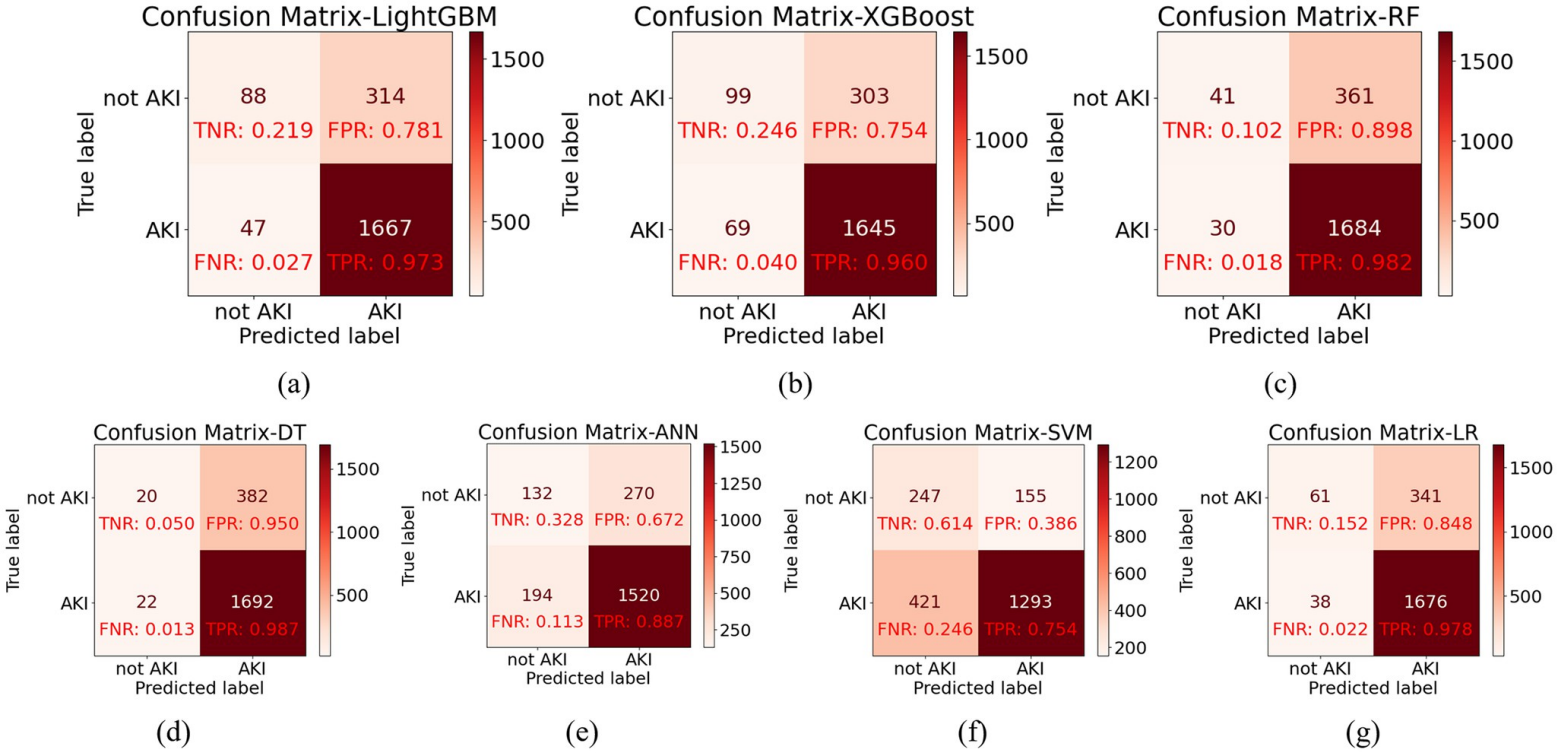
Confusion matrix for 7 models. (a) LightGBM (b) XGBoost (c) RF (d) DT (e) ANN (f) SVM (g) LR.

Furthermore, Fig 3 depicts the confusion matrices of the various models, providing additional insights. This suggests that the LightGBM model excels in detecting positive samples, showcasing improved sensitivity—an essential aspect for early AKI patient identification. In summary, it evidences superior performance in predicting AKI patients.

**Fig 4:**
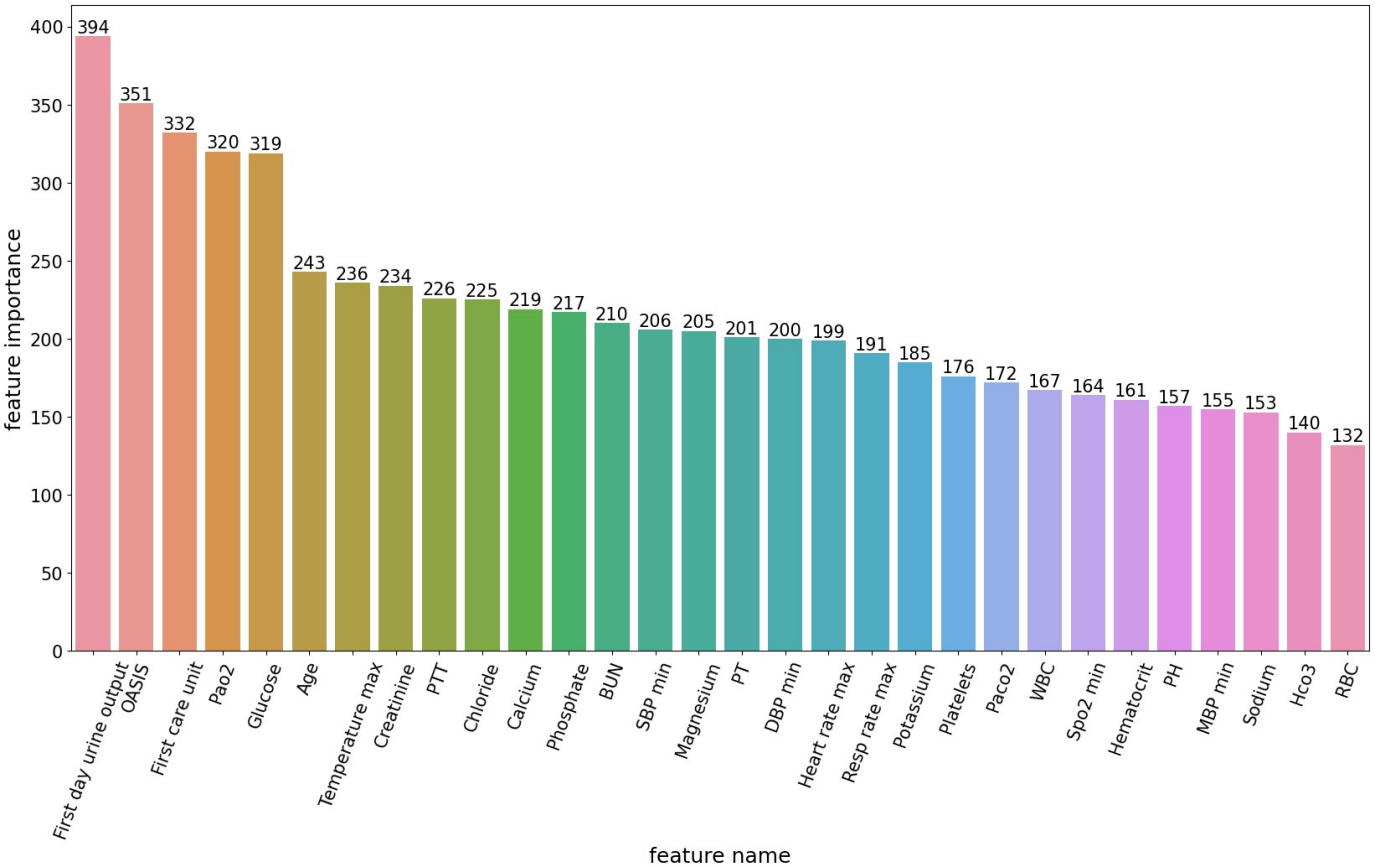
Top-20 variable importance of LightGBM.

Finally, an analysis of feature importance in the LightGBM model was conducted, revealing the top-ranking features in descending order. As illustrated in Fig 4, the most critical predictors included first day urine output, OASIS, first care unit, Pao2 and glucose, which contribute to higher predictive powers than the bottom features. This indicates that these features contribute significantly to the prediction results. In the next section of the discussion, a more in-depth analysis of the AKI diagnosis problem will be conducted, potentially uncovering new correlations and patterns.

## 4 Discussion

Sepsis, a prevalent critical condition in the ICU, stands as a primary cause of mortality among critically ill patients globally. Within the realm of critical care medicine, sepsis remains a challenging and extensively studied subject. Its intersection with acute kidney injury (AKI) presents as a common clinical complication in patients diagnosed with sepsis. Timely detection and proactive early active treatment can significantly improve the survival rate of AKI patients. Hence, the development and promotion of responsible AKI predictive models for sepsis patients is of great significance in improving patient prognosis. Based on the experimental findings, the following conclusions can be drawn:

Firstly, from the Table 3, it can be observed that the LightGBM model’s superior performance, boasting the highest AUC (0.801), accuracy (0.829), and F1 score (0.902) in predicting AKI among patients diagnosed with sepsis. This robust performance indicates its exceptional capability in discriminating between positive and negative instances. Such findings suggest the model’s effectiveness in accurately ranking samples and allocating higher probabilities to positive cases, thereby enhancing decision-making processes. However, it’s noteworthy that SVM exhibits exceptional specificity and precision. Researchers and practitioners might consider selecting models based on specific task requirements.

Moreover, the discussion encompasses 10,575 critically ill patients diagnosed with sepsis, among whom 8,575 experienced AKI during their hospitalization. This incidence rate of AKI among the patients studied herein notably surpasses the reported 40%-50% in relevant literature. Several factors may contribute to this disparity: (1) The study being a retrospective real-world investigation in a single center, where disease incidence is influenced by the center’s diagnosis and treatment standards; (2) The inclusion criteria encompass all sepsis patients, irrespective of AKI status or clinical staging. Experimental outcomes demonstrate the LightGBM model’s exceptional predictive probability calibration, closely mirroring actual probabilities. Even in the face of an imbalanced distribution between positive and negative instances, the model adeptly distinguishes between the two, displaying remarkable robustness in managing class imbalances.

Secondly, from Fig 4, it can be seen that the top 5 important features were first day urine output, OASIS, first care unit, Pao2 and glucose, which contribute to higher predictive powers than the bottom features. Among these features, urine volume, is the most significant index for predicting AKI, conforming to the suggestions of the KDIGO guidelines. A decrease in urine output can be an early sign of AKI. OASIS is a scoring system that evaluates the severity of illness in patients, which can provide valuable information about the overall health status of a patient, including the presence or risk of AKI. The location or type of care unit where a patient is admitted can be an important factor in identifying AKI patients. Certain care units, such as ICU, are more likely to have patients at higher risk of developing AKI due to the severity of their illness or the nature of the treatments they receive. Pao2 reflects the oxygenation status of the blood. In patients with AKI, there may be reduced renal blood flow and impaired oxygen delivery to the kidneys. This can result in tissue hypoxia and further damage to the kidneys. Low Pao2 levels can indicate the presence of respiratory dysfunction or lung disease, which can contribute to the development or progression of AKI. Abnormal glucose levels, especially high levels (hyperglycemia), can contribute to the development or worsening of AKI. Hyperglycemia can cause inflammation, oxidative stress, and endothelial dysfunction, all of which can damage the kidneys. Monitoring and assessing these five important features can help in the early identification of AKI patients.

Thirdly, LightGBM offers a feature importance ranking, aiding in the identification of the most impactful features and providing insights into the model’s predictions. This functionality empowers clinicians to recognize factors contributing to AKI prediction, enabling them to prioritize interventions. Adjusting medication dosages, optimizing fluid management, or implementing renal protective strategies based on identified risk factors can significantly influence patient outcomes.

Furthermore, machine learning algorithms like LightGBM demonstrate significant capability in managing extensive datasets and enabling real-time information processing, rendering them highly compatible with integration into electronic health record (EHR) systems. This integration harbors the potential to establish an automated system capable of predicting AKI risk and generating alerts, thereby providing clinicians with timely notifications and empowering proactive patient care.

Nevertheless, it’s crucial to recognize several limitations in our study. The retrospective observational design introduces the potential for selection bias. The dataset used was sourced from a single center in the United States, casting doubt on the generalizability of our findings to a broader population. Thus, extensive large-scale, multi-center studies are warranted to confirm the model’s suitability across diverse settings. Furthermore, further research and validation in real-world clinical environments are essential to comprehensively evaluate its practical impact and feasibility for widespread adoption.

## 5 Conclusion

In conclusion, our study demonstrates the efficacy of machine learning models in accurately predicting AKI among septic patients. Among the diverse predictive models assessed, LightGBM stands out with the highest predictive performance. This model holds promise as a dependable tool for healthcare practitioners to identify high-risk patients and tailor personalized treatment strategies. Implementing early interventions for patients diagnosed with sepsis at AKI risk could potentially reduce mortality rates, although this warrants further prospective research for confirmation.
